# Anæsthetics
*A Paper read at a Meeting of the Bristol Medico-Chirurgical Society held at the University on 8th May, 1929.


**Published:** 1929

**Authors:** Stuart V. Stock

**Affiliations:** Lecturer in charge of the Anœsthetic Department, the University of Bristol; Honorary Anœsthetist, Bristol Royal Infirmary


### ANÆSTHETICS.*

BY

STUART V. STOCK, M.B., B.S., M.B., B.CH., F.R.C.S.,
*Lecturer in charge of the Anæsthetic Department, the University of Bristol;*
*Honorary Anæsthetist, Bristol Royal Infirmary.*

---

WITHOUT modern methods of asepsis, antisepsis and anæsthesia modern surgery would be impossible. This statement cannot be challenged, and so anæsthesia has assumed an importance formerly lacking.

That it is as yet far from perfect a glance at the following figures will show. These as nearly as possible express the present *immediate* mortality rate from the anæsthetics named :—

Chloroform.—1 in 2,000–3,000 cases.

Ether.—1 in 16,000.

Nitrous Oxide and Oxygen. — The immediate mortality in minor surgery is practically nil, but in major surgery it is about the same as in ether (1 in 16,000).

When analgesic methods are used, the only available figures are for spinal block; these vary enormously according to the method, the experience and veracity of the administrator.

I have analysed about 45,000 cases, and find that the mortality works out at 1 in 770 cases. This figure,

* A Paper read at a Meeting of the Bristol Medico-Chirurgical Society held at the University on 8th May, 1929.

which is rather appalling, included the stovaine deaths. Stovaine, like chloroform, should never be used. This is not the whole tale, because there are late effects producing fatalities which are not included above.

At the present time the anæsthetist holds a well-defined and I hope well-merited place in the surgical team as the surgeon's chief assistant, with definite duties and responsibilities, one of the most important being his examination of the patient, to estimate his fitness for operation and to choose the best method of anæsthetising him, both from his own and the surgeon's point of view. "Operation successful, result death," is a jibe that has often been hurled at surgical procedures in the past, and it behoves the anæsthetist to see that he does not contribute to this consummation.

It is useless to state that a patient is fit to take an anæsthetic and to leave the matter there; what is wanted is an estimate of the patient's fitness to stand an operation.

As the late S. R. Wilson[1] has pointed out, besides the ordinary examination of the heart, lungs and urine, the estimation of the blood-pressure affords the most valuable information in relation to operative prognosis. It enables one to determine the condition of the cardiac muscle and indicates the capacity of the patient to withstand hemorrhage, shock and surgical manipulation. It also modifies the choice of the anæsthetic. The factors required are the pulse-rate, the diastolic and systolic pressures and their difference, which is the pulse-pressure.

From these we get two formulæ, worked out by Moot and McKesson, which are called the *pressure ratio* and the *energy index*.

The pressure ratio $= \frac{\text{pulse-pressure}}{\text{diastolic pressure}} \times 100$. If the resulting percentage lies between 30 and 70, the case is considered a favourable one. It has been pointed out that in both aortic regurgitation and hyperpiesis the figure obtained by this formula lies outside the safe limit laid down, but that cases of this description take anæsthetics well. This I grant, but the indication we seek is the patient's capacity to stand hemorrhage, shock and surgical manipulation, not his ability to inhale safely a certain amount of anæsthetic. I have applied this test to every doubtful case handled during the last two years, and have been helped over and over again. It is not intended to imply that cases showing a percentage figure outside these safe limits will not stand a severe operation, but that they require very much greater care; and a more guarded operative prognosis must be given. Into this category fall our cases of hyperpiesis and aortic regurgitation.

The second of the above two formulæ, the "energy index," is obtained by adding the systolic pressure to the diastolic pressure and multiplying by the pulse-rate. In the resulting answer we ignore the hundreds, and take only the figure representing the thousands. This should lie between 12 and 18.

The following example will show what is meant:—

In a patient with systolic pressure of 120 and a diastolic of 80, and a pulse-rate of 72, the energy index is $(120 + 80) \times 72 = 14{,}400$. We disregard the 400 and take the figure 14. This, lying between our 12 and 18 limits, indicates that the case is a favourable one.

I have used this formula in a large number of cases, but have never got the same help as from the "pressure ratio." Of this I can offer no explanation.

In operations of any magnitude it is of the utmost assistance to have a record of the blood-pressure at, say, 5 to 10 minutes' intervals, together with the pulse-rate. A rise in the pulse-rate always precedes a fall in the blood-pressure, while a drop in the systolic pressure accompanied by a *rise* in the diastolic pressure is a sign of grave omen necessitating prompt measures to restore the pressures to a more normal relationship. A very low systolic blood-pressure is of itself not necessarily fatal, as I have under spinal anæsthesia seen pressures of 75 and 80 m.m. of mercury without the patient showing much general change. In both cases the pressure quickly rises again to well above the 100 mark.

In preparing patients for operations there are several things to be kept in mind, but, as F. J. McCann has pointed out, the chief is to disturb the patient physically or mentally as little as possible. Every operation is an ordeal of greater or less severity, which may leave a lasting impression on its subject. The late Professor Michell Clarke told me he considered that after a big operation it took two years before a patient returned to the normal condition. Modern technique has shortened that time, but even now it is a matter of months rather than weeks before all trace of the ordeal is lost. In this connection too much importance cannot be ascribed to the work of Crile on the avoidance of psychic and traumatic shock.

Forty-eight hours in a nursing home or hospital before operation is essential if it can be managed. The patients always do better both at the time of operation and afterwards. I have never yet met a sister or nurse of large experience who was not emphatic on this point.

The old ideas of starvation and purgation still linger. There is no worse preparation, and the enema before operation, unless specially indicated, should be abolished. Castor oil, if the patient is constipated, is all that is required. As McCann says : " To give a purgative the previous night to a woman about to undergo an abdominal operation, and to awaken her at 5 a.m. for an enema and a cup of tea when the operation is to be performed at 8.30 or 9 a.m., is a form of scientific cruelty which should be abolished."

It should never be forgotten that an enema may cause considerable shock and distress to the patient.

The patient should take his ordinary meals, up to and including lunch on the day before operation ; after that, a light tea and an easily digestible supper of small dimensions is usually all the starvation required. Sleep the night before an operation is of the very greatest importance, and if there is any doubt about it an hypnotic should be given, but it must be adequate. My own preference is for bromidia.

In bad risk cases glucose before operation is a great help ; it does not matter much how it is administered, though if possible I prefer to give it by mouth. A clever nurse with a little imagination will help one greatly in this respect.

In my opinion there is no evidence of the value of rectal salines before operation, except in special cases. If fluid is wanted let it be given by the mouth when the patient can take it.

In these bad risk cases I have been employing for some time now a pre- and post-operative treatment with ephedrine, which was first suggested to me by my colleague Dr. A. T. Todd, and it is no exaggeration to say that the results obtained have been remarkable. Shock has been abolished or so largely reduced as to be of little or no importance, but the technique must be strictly followed. It consists of giving the patient by mouth a mixture containing ephedrine gr. $\frac{1}{3}$, tincture of digitalis m. vii., and mist. calcii albuminat. ℥i. The calcium albuminate takes some little time to prepare, and the pharmacist cannot produce it at once. Five doses of this are given at four-hourly intervals, the last dose an hour before operation. A similar number of doses is given after operation. If, owing to post-operative sickness, it cannot be given at first by the mouth, the ephedrine should be given hypodermically, *combined with caffein,* according to the following formula which we use at the Bristol Royal Infirmary :—

Ephedrine hydrochlor. gr. $\frac{1}{3}$ ; caffein, gr. 2 ; sodium salicylate, gr. 3 ; glycero-alcohol, min. 16.

Ephedrine should never be given by itself, for some people have an idiosyncrasy for this drug. It acts on the vasomotor nerves, and as its effect passes off, what may be called a negative phase sets in. The vessels dilate, the cardiac contractions become less forcible ; at this point the vagus action on the heart seems to be stimulated, leading to slowing of the heart and circulatory collapse. Caffein will prevent this, as it carries the circulation over the negative phase. Digitalis and calcium have the same action, but the caffein was chosen as it could be given hypodermically.

Fortunately, these susceptible people are rare, but

I have seen two cases of circulatory collapse after operation in which ephedrine alone was used.

Only two minor drawbacks to this method of treatment have so far been noted. The first is that during operation a few patients have sweated rather profusely, so that to prevent chilling care must be taken to see that the patients are properly covered. The other is that after operation the excretion of urine may be diminished. This was only marked in one case, and stopping the mixture promptly restored the excretion to normal.

The number and strength of the doses given above should be considered as maximum. The larger amounts of ephedrine given by some of the American surgeons are neither advantageous nor desirable. Our aim should be to "prop up" the circulation to a point that will carry it through the strain of the operation with the minimum effective dose of the drug possible.

Before leaving this subject, I should like to express my indebtedness to Dr. Todd for the help and advice he has given me in the working out of the technique.

The preliminary medication of the patient has been much debated, but with inhalation anæsthetics atropine is essential. Introduced at first to prevent reflex cardiac inhibition through the vagus, it has now the definite object of controlling oral and bronchial secretion. The dose used varies. I prefer 1/75 grain three-quarters of an hour before operation in the case of adults. Children over twelve are given 1/100 grain, and below that age in proportion. Young children have it by mouth. The dose that will suit all cases does not exist, as the dose of atropine is an individual one.

As the preliminary use of morphia, omnopon and hyoscine modifies the usual signs of anæsthesia, special care is required when employing them. Of their advantage there is no question, but certain disadvantages have to be considered. They protect the patient to a large extent from psychic shock, diminish the amount of anæsthetic required, and in this way help to guard against traumatic shock. By cutting down reflex excitability they make it possible to anæsthetize for many operations with gas and oxygen, that would otherwise require the addition of ether.

Some anæsthetists use the morphia-hyoscine, others the omnopon-hyoscine combination. For uniformity of effect the ampoules of scopolamine-omnopon Roche I have found most reliable, given at least one hour before operation. Even two hours before will be found advantageous in some cases.

When either combination is used, the patient should be placed on the theatre trolley at the time of injection, and left quietly in a darkened room until the operation. Failure to observe this rule destroys half the advantages of the injection. The patient is on no account to walk to the theatre after either of the above drugs has been given.

The disadvantages are few but marked: dryness of the mouth before operation and in some cases increased vomiting after; increased rigidity of abdominal walls during operation has been claimed as due to the combination, and there is no doubt that in many inhalation cases the time of induction is prolonged.

During operation the effect of the morphia on the respiratory centre may give rise to trouble unless care is taken, and on this account chloroform, except in very small quantities, should never be used.

Finally, hyoscine causes an increase of bleeding, owing to dilatation of the vessels ; so much so that for years I have given it up in operations on the throat and nose. Vomiting after either morphia or omnopon may be marked ; in my experience more so after omnopon than morphia. This is disappointing, and I have been at some pains to assure myself that my first impression was true, as the primary effects of the omnopon and hyoscine are so good. For the thirst water or lemonade should be given, unless the operation is on the stomach or duodenum. My patients get as much as they want, and I have never been sorry for giving it.

After these drugs the patient must be watched a little longer than usual.

Before considering the choice of a method for actually anæsthetizing the patient, let us face the fact that whatever anæsthetic or analgesic agent is used it is a potential poison, and that we as anæsthetists are potential poisoners. The idea that any anæsthetic agent is harmless is wrong ; none are, though some are more poisonous than others. From this it follows that our object should be to give as little of the drug as we can, always remembering that our anæsthesia or analgesia must be adequate. We must always recollect that adequate anæsthesia may be deep or light. No more pernicious doctrine exists in medicine than the teaching that a patient must be reduced to a certain depth of anæsthesia and kept there, irrespective of the surgical need of the moment. Neither is it right to say that a semi-conscious patient is a suitable subject for an abdominal operation, and that the surgeon must learn to operate on lightly-anæsthetized subjects. Adequate anæsthesia gives us our desired effect with the least possible expenditure of anæsthetic, and is nicely adjusted to the needs of the moment, not to what was wanted five minutes ago or will be at some time ahead. Adequate anæsthesia is the best thing for the surgeon and therefore for the patient, always remembering that the anæsthetist is there to assist the surgeon, thus helping him in every possible way, unless he be of the variety who requires his patients so deeply under that they get to the fourth stage of anæsthesia. These profound anæsthesias, soaking the patient with ether, are chiefly responsible for the damage done by inhalation anæsthetics.

In choosing the actual method, what we have to consider is the patient, and not which drug shall be used. In doing so there are three factors to consider : psychic shock, traumatic shock, and the toxic effect of the anæsthetic. The majority of patients met with in this country ask: "Let me know nothing about it." This practically means an inhalation anæsthetic of some sort, and in choosing it there is one drug that should never be used now, *i.e.* pure chloroform. In my opinion chloroform alone should only be used for diathermy operations about the mouth, and even then a mixture of chloroform and ether can be used for the induction.

Another " don't " is, never give pure ether to a case of phthisis ; the risk of causing an exacerbation of the condition is very great.

For the man who does not give many anæsthetics, and who wants a method that is generally applicable, the light ether anæsthesia, which A. L. Flemming has done so much to popularize, cannot be beaten. A Shipway's apparatus should be used, with the tube leading from it not more than three feet long. The chloroform bottle, if it has one attached, is ignored. Anæsthesia is induced with the ordinary mask, and when the Shipway is turned on the least amount of ether effective is used. Outside the abdomen and the mouth, gas and oxygen, together with morphia and perhaps a very little ether, constitute an ideal anæsthetic, with one drawback, that it is the most difficult of all the anæsthetics to give.

For abdominal work I pin my faith at present to a combined method of local and general anæsthesia ; an induction by a few drops of chloroform followed by ether, infiltration of the muscle of the abdominal wall, and of the mesentery of the organ or bowel to be operated on, followed by gas and oxygen for the operation itself. The peritoneum is closed under a little C.E. mixture followed by gas and oxygen. At the end the patient's lungs are thoroughly "washed out" with 5 per cent. carbon dioxide in oxygen.

As this paper deals rather with anæsthetics than analgesics, I will only add that if a regional anæsthetic is used, such as spinal, or some other nerve-blocking or local procedure, it is essential to see that the patient is rendered amnesic by morphia and hyoscine, gas and oxygen, or even a little ether, otherwise a condition of psychic shock may be produced that will have a lasting effect on the patient.

As to the actual administration of the drug itself, there are two golden rules to follow. The first is, maintain the air way. I believe that no case of death under ether has been recorded where the asphyxial element was absent. Secondly, establish and maintain automatic respiration with the smallest amount of the anæsthetic possible, using the eye reflexes only when in doubt as to the depth of anæsthesia, *i.e.* the eye reflexes are to be a check on, and not a guide to, the depth of anæsthesia. The character of the respirations are the most delicate guide of all, and any time and trouble spent in mastering them will be repaid manifold.

Finally, a few remarks on post-anæsthetic toxæmias may not be out of place.

The late S. R. Wilson published a most excellent article[1] on these. They may occur after all general anæsthetics, even gas and oxygen, but most commonly and severely after chloroform, which has a definite action on the heart muscle, liver and kidneys, producing granular swelling and fatty degeneration.

There are three types of toxæmia, the cardiac, renal, and hepatic. The hepatic type is the commonly recognized variety. The renal type is usually transient, but in rare cases may pass into urinary suppression and uræmia. The cardiac type is most important, and explains many cases of death after operation, deaths which are usually ascribed to anything but their true cause, the anæsthetic.

Wilson pointed out that the symptoms are an acceleration of the pulse with more or less normal respiration, vomiting varying with the extent of the associated hepatic involvement, physical signs of cardiac dilatation and sudden or gradual heart failure. Thrombosis and embolism may occur as a result of the cardiac enfeeblement.

### DISCUSSION.

DR. C. CORFIELD agreed that the fitness of a patient to stand the operation was the important point, rather than the fitness to stand the anæsthetic. On such a subject the anæsthetist was well qualified to express an opinion—he knew the surgeon and the proposed operation, and had accumulated a large experience on this very point, how operations affected patients. He wished to endorse what Mr. Stock had said as to the value of atropine, and liked a full dose, *e.g.* gr. 1/75 for an ordinary adult. Morphine he did not like ; it lowered the blood-pressure.

DR. C. E. K. HERAPATH criticized the " pulse ratio and energy index method " of estimating the prognosis for anæsthesia, and gave examples of frequent heart lesions which did not at all form contra-indications, but which by that method would " bar " operations. He felt neurasthenics should avoid all but urgent operations. Some patients, he would point out, had an idiosyncrasy for atropine, the usual dose producing among other symptoms intense tachycardia. It was news to him that gas and oxygen could upset a patient—was the effect of a ketosis rather exerted on the medulla than on the heart ?

DR. F. H. BODMAN said that he personally found it important to forbid smoking immediately before an operation. As a preliminary medication he preferred an injection of morphine with scopolamine. He had tried the " energy index " method but did not find it of value

DR. H. B. LOGAN spoke of the importance of fear before an anæsthetic, and especially fear of the anæsthetic and fear that "they will start before I'm under." He employs a bromide and chloral mixture the night before the operation. He regularly uses hyoscine as a preliminary injection, but does not like morphine.

Mr. L. E. Claremont spoke of the distress sometimes seen with analgesia, and inquired whether it was psychological or possibly due to use of too much adrenaline.

Dr. L. A. Moore said he thought hyoscine increased the difficulties of induction, and had no compensatory advantages.

REFERENCE.

[1] S. R. Wilson, *British Journal of Anæsthesia,* 1925, Vol. iii., No. 2, p. 6; 1926, Vol. iii., No. 3, pp. 49, 126.

